# Design of Waveguide Polarization Convertor Based on Asymmetric 1D Photonic Crystals

**DOI:** 10.3390/nano12142454

**Published:** 2022-07-18

**Authors:** Fu-Li Hsiao, Chia-Ying Ni, Ying-Pin Tsai, Ting-Wei Chiang, Yen-Tung Yang, Cheng-Jui Fan, Hsuan-Ming Chang, Chien-Chung Chen, Hsin-Feng Lee, Bor-Shyh Lin, Kai-Chun Chan, Chii-Chang Chen

**Affiliations:** 1 Institute of Photonics, National Changhua University of Education, Changhua 500, Taiwan; fulihsiao@cc.ncue.edu.tw (F.-L.H.); d0526004@mail.ncue.edu.tw (C.-Y.N.); d0926004@mail.ncue.edu.tw (T.-W.C.); d0926002@mail.ncue.edu.tw (Y.-T.Y.); d0926003@mail.ncue.edu.tw (C.-J.F.); d0926001@mail.ncue.edu.tw (H.-M.C.); james586509@gmail.com (C.-C.C.); hsinfeng.lee0616@gmail.com (H.-F.L.); 2 Institute of Imaging and Biomedical Photonics, National Yang Ming Chiao Tung University, Tainan City 711, Taiwan; yingpintsai@gmail.com (Y.-P.T.); borshyhlin@nycu.edu.tw (B.-S.L.); 3 Department of Optics and Photonics, National Central University, Taoyuan City 320, Taiwan; zxc7453708@gmail.com

**Keywords:** integrated optics, optical polarization, photonic crystals

## Abstract

Photonic crystals possess metastructures with a unique dispersion relation. An integrated optical circuit plays a crucial role in quantum computing, for which miniaturized optical components can be designed according to the characteristics of photonic crystals. Because the stable light transmission mode for a square waveguide is transverse electric or transverse magnetic polarization, we designed a half-waveplate element with a photonic crystal that can rotate the polarization direction of the light incident on a waveguide by 90°. Using the dispersion relation of photonic crystals, the polarization rotation length and the optical axis’s angle of deviation from the electric field in the eigenmode can be effectively calculated. Polarization rotators designed on the basis of photonic crystal structures can effectively reduce the insertion loss of components and exhibit favorable polarization rotation performance.

## 1. Introduction

Photonic crystals, which possess an artificial periodic structure, have a unique dispersion relation and are used extensively in the design of optical components, such as optical lenses [1,2,3,4], optical waveguides [5,6], and optical waveplates [7,8,9]. Photonic crystals with different structural designs can be exploited to tailor the dispersion relation and spatially exhibit different effective refractive indices of guided modes in the waveguide.

Integrated optical circuits [10,11] are expected to play a crucial role in the development of quantum computers. A complete integrated optical circuit for quantum computing can be easily fabricated on a chip by using a miniature optical structure [12,13,14,15]. In the optical quantum computing design, silicon may be a good material candidate at a certain low operating temperature due to the stable dispersion relation [16]. Because of the inherent properties of photons, the polarization state of light can be considered a quantum mode in quantum calculations. Two orthogonal polarization modes can be selected as optical quantum modes, and a polarization rotator can be used to switch between [15] or facilitate the entanglement of [12] these quantum modes.

Miniaturized optical components built into waveguide-type structures are connected with other fibers or waveguides and integrated into the optical circuit on a chip. Currently, waveguides are commonly produced with square cross sections; therefore, the light waves transmitted in waveguides exhibit transverse electric (TE) or transverse magnetic (TM) polarization. Most relevant studies have used elements with asymmetric cross sections to achieve conversion between these polarization modes, with the polarization rotation effect being produced by the difference in the transmission constants between these modes. Common methods used to achieve polarization rotation include embedding the main waveguide in the corner of the outer waveguide [12,17], digging a groove on one side of a waveguide [18,19], cutting one side of a square waveguide into an oblique shape [20,21,22,23], and developing a waveguide with an L-shaped (asymmetric) [24,25] or double-stair cross section [26].

One particular avenue that was recently proposed in this context is using the photonic crystal [27]. Chen designed a polarization rotator based on one-dimensional photonic crystals. In addition to using arrays of air cylinders to produce asymmetric cross sections, they used photonic crystals to design a new type of polarization rotator with high performance and potential.

In this study, we designed a waveguide-type wave plate element with one-dimensional photonic crystals. We formed an asymmetric waveguide cross section by placing periodic arrays of air cubes on a square Si waveguide to create two polarization modes with different effective refractive indices of the guided mode in the waveguide (n_f_ and n_s_). The angle between the optical axis and the coordinate axis of the two modes in the crystals was 45°, enabling the polarization angle to be rotated by 90° beyond a certain length (*L*_π_) in the wave plate element to achieve TE–TM conversion. We calculated the polarization rotation length *L*_π_ by analyzing the photonic crystal band structure and determined the angle between the optical axis and the coordinate axis (optical axis deviation angle) by using the horizontal and vertical components of the electric field in the eigenmode. Changes in the geometric parameters of the air cubes can cause changes in the deviation angle, effective refractive index, and insertion loss (*IL*) in the eigenmode. Therefore, when maintaining *L*_π_ between 2.5 and 3 μm, we adjusted the geometric parameters of the air cubes to reduce the *IL* and thus improve the performance of the structure of the wave plate element.

## 2. Materials and Methods

**Figure 1:**
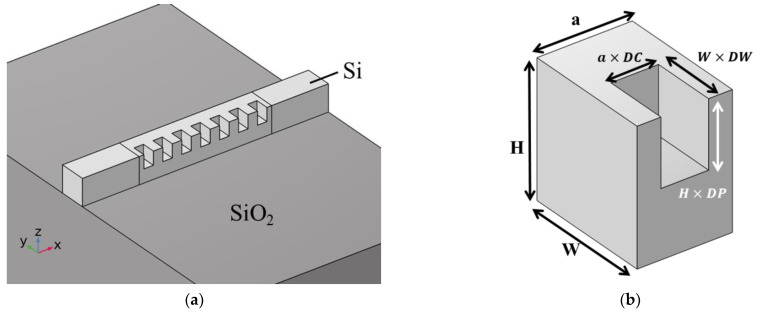
Schematics of the (**a**) full structure and (**b**) unit cell of the photonic crystal waveplate.

A schematic of the structure of the photonic crystal waveguide is displayed in Figure 1. We used an air cube array on one side of the square waveguide to form a tooth-shaped waveguide, as shown in Figure 1a. A SiO_2_ substrate (dark gray area in Figure 1) was placed below the waveguide. The waveguide (light gray area in Figure 1) consisted of square waveguides at the front and back for guiding electromagnetic waves and a tooth-shaped multi-period waveguide. These waveguides were made of Si and placed beneath an air layer that occupied the space above (not illustrated here). The refractive indices of SiO_2_, Si, and air were set as 1.46, 3.46, and 1, respectively. Figure 1b depicts a unit cell and the detailed structural parameters of the periodic tooth-like structures, where *a* is the lattice constant, *H* is the height of the waveguide, and *W* is the width of the waveguide. We defined the thickness, depth, and width of the air cube in the waveguide as *a × DC*, *H × DP*, and *W × DW*, respectively. To maintain the asymmetry of the waveguide’s cross section (similar to an L-shape), we limited *DC*, *DP*, and *DW* to 0.2–0.8.

We used the finite-element method (FEM) to simulate the light transmission behaviors and the photon band structures in the full structure. Band structures and light transmission behaviors were determined using eigenmode analysis and frequency–domain analysis, respectively. In the calculation of the eigenmode, we only used a unit cell with a width of *a* (Figure 1b). The band structure diagram of the photonic crystal was calculated by setting Floquet periodic boundary conditions in the x-direction, and the deviation angle of the optical axis of the wave plate was determined from the electric field data for the eigenmode. In addition, the polarization transition state and *IL* values of the full structure were analyzed in the frequency domain by plotting the full structure of the waveplate (Figure 1a). This structure contained multi-period tooth-shaped waveguides and complete square waveguides at its head and tail. An electromagnetic wave with a wavelength of 1.55 μm and y-polarization was incident onto the square waveguide from the x-direction, and it entered the tooth-shaped waveplate structure after passing through a complete square waveguide.

## 3. Results

**Figure 2:**
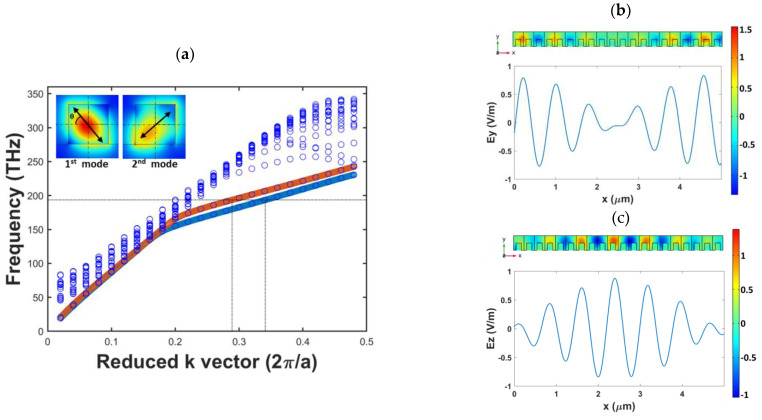
(**a**) Band structure diagram (the insets display the electric field distributions of the first and second modes), (**b**) distribution and intensity maps of the y-component electric field, and (**c**) distribution and intensity maps of the z-component electric field of the structure with *DC* = 0.5, *DP* = 0.5, and *DW* = 0.5.

We first selected the structure with *DC* = *DP* = *DW* = 0.5 for analysis and used the unit cell displayed in Figure 1b to obtain the band structure diagram of the photonic crystal through FEM-based eigenvalue calculation (Figure 2a). The horizontal axis of the band structure diagram in Figure 2a represents the reduced k-vector. The unit of this vector is 2π/a, and it represents the reciprocal space coordinates in the reduced Brillouin zone. The vertical axis of the aforementioned diagram denotes the eigenfrequency. The points in Figure 2a, which carry different k-vectors, represent the eigenfrequencies at specific k vectors. By connecting those points with similar field distributions, we found several lines in the band structure diagram, which represent the eigenmodes existing in the corresponding structure. We increased the number of data points in the two modes with the lowest frequency by using the interpolation method, and these modes are indicated using blue and orange lines in Figure 2a. The operating frequency was set as the frequency corresponding to a light wave with a vacuum wavelength of 1.55 μm (approximately 194 THz), which is marked with a dotted line Figure 2a. The photonic crystal structure corresponds to the two eigenmodes at the operating frequency, and the k-vectors in these eigenmodes had values of 0.289 (second mode, orange line) and 0.341 (first mode, blue line). After substituting the lattice constant *a* and wavelength into the reduced k-vector unit noted previously, the refractive index of the first and second modes were obtained (n_s_ = 2.114 and n_f_ = 1.792). We defined the optical axis of the first mode as the slow axis and that of the second mode as the fast axis. The electric field diagrams of the two modes are presented in the insets of Figure 2a.

Because the wave incident from the square waveguide exhibits TE or TM polarization, the half-wave-plate conditions must be fulfilled to achieve conversion between the two orthogonal linear polarization modes. According to the birefringence properties of a half-wave-plate, a phase difference $\Gamma =\frac{2\pi \cdot \Delta n\cdot L}{\lambda_{0}}=\pi$ exists between the components of the incident wave transmitted along the fast and slow axes. In the aforementioned equation, Δn represents the differences in the refractive indices of the fast and slow axes, *L* represents the polarization rotation length, and $\lambda_{0}$ represents the wavelength of an incident wave in vacuum. According to the formula, the polarization rotation length can be found that is inverse proportional to Δn at certain wavelength. Therefore, increasing the difference of the refractive indices between eigenmodes can produce shorter *L*. After substituting the two effective refractive indices of the guided modes and the vacuum wavelength of 1.55 μm into the aforementioned equation, the required polarization rotation length for the photonic crystal half-wave-plate was obtained as 2.41 μm (*L*_π_ = λ_0_/(2∙∆*n*) = 2.41 μm). It can be noticed that it should be a reciprocal result when the light comes from the other side of the device due to the same modes. To verify this result, we performed frequency–domain analysis with a 20-period full structure. The electric field distribution and electric field intensity obtained when a y-polarized electromagnetic wave was transmitted along the waveguide are illustrated in Figure 2b,c, respectively. The electric field intensity map in the lower parts of Figure 2b,c reveals that *E_y_* (intensity along the y-axis) was almost 0 at *x* = 2.45 μm. Moreover, at this point, *E_z_* (intensity along the z-axis) reached its maximum value. The aforementioned results indicate that the analyzed structure converted y-polarized waves to z-polarized waves at a minimum distance of approximately 2.45 μm, which is close to the theoretical value obtained using the photonic band structure diagram.

For better understanding of the birefringence properties of a PhC-type wave plate structure, we calculated the optical axis deviation angle of the photonic crystal wave plate. By using the y- and z-component strengths of the electric field of the eigenmode to integrate the waveguide cross section Ω, the angle *θ* between the optical axis and the coordinate axis of mode can be defined as follows [19]:

(1) $$\tan\theta =\frac{\iint_{\Omega }^{}n_{Si}{}^{2}\left(y,z\right)\cdot E_{z}{}^{2}\left(y,z\right)dydz}{\iint_{\Omega }^{}n_{Si}{}^{2}\left(y,z\right)\cdot E_{y}{}^{2}\left(y,z\right)dydz}$$

where *n_Si_* is the refractive index of *Si* (i.e., 3.46).

**Figure 3:**
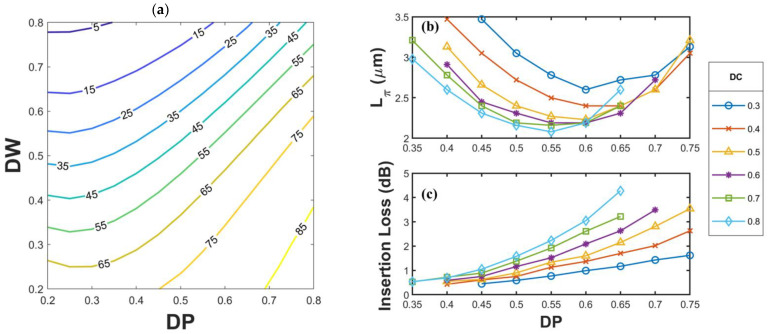
(**a**) Contour map of the optical axis deviation angle when DC = 0.5, (**b**) polarization rotation length, and (**c**) *IL* when *θ* = 45°.

The inset of Figure 2a shows the optical axis deviation angle *θ* in the first mode when *DC* = *DP* = *DW* = 0.5. Using Equation (1), we obtained θ to be 49.7°. Because of the presence of a SiO_2_ substrate under the waveguide, even when the cross section of the waveguide had a symmetric pattern with x = y as the axis of symmetry, the asymmetric material distribution around the waveguide caused the included angle of the optical axis to not be 45°. According to the characteristics of a half-wave-plate, when the phase difference between the waves passing along the fast and slow axes of a birefringent element is π, the angle φ between the original polarization direction and the optical axis is rotated to 2φ after these waves are linearly polarized and pass through the element. To achieve conversion between TE and TM polarization, 2φ should be 90°; therefore, a birefringent structure should be designed to have a φ value of 45° (i.e., *θ* = 45° is angle between the optical axis and the coordinate axis). To design a structure with *θ* = 45°, we calculated the optical axis deviation angle *θ* for the first mode according to the structural parameters *DC*, *DP*, and *DW*. Figure 3a displays the contour map drawn for *DC* = 0.5. The gradient of the contours in this figure exhibits an increasing trend from the upper left to the lower right, and the 45° contour is very close to the diagonal where *DP* = *DW*. The aforementioned figure reveals that when *DP* < 0.4, the slope of the contours gradually decreases, and the gradient distribution gradually shifts to the vertical direction; when the *DP* value is changed, the *DW* difference corresponding to the same optical axis angle is not very large. The 45° contour line has a high overlap with the *DP* = *DW* line only when *DP* > 0.6.

We extracted the part with $L_{\pi }<3.5$ μm from all the *DP* and *DW* combinations on the 45° contour line with different *DC* values, and the corresponding *DC*, *DP*, and *DW* values are listed in Table 1. On the basis of the structural parameters presented in Table 1, *L*_π_ and *IL* were plotted for different components (Figure 3b,c). The term *L*_π_ was calculated using the refractive index difference obtained through eigenvalue analysis. To determine the *IL*, tooth-shaped structures with lengths corresponding to the lengths of each structure (*L*_π_) were drawn (e.g., for *DC* = 0.4, *DP* = 0.4, and *DW* = 0.46, *L*_π_ = 3.47 μm, which is approximately 13.88 times the value of *a* (=0.25 μm), and the tooth-shaped structure was drawn for 14 periods). The connected square waveguides in the front and back side of the tooth-shaped region are five periods long, and a y-polarized electromagnetic wave was incident from the x-direction of the square waveguide. The *IL* can be obtained from the output signal *E_out_* and incident signal *E_in_* received from the output end of the square waveguide as follows:

(2) $$IL\left(\mathrm{dB}\right)=10\times \log_{10}\frac{E_{in}}{E_{out}}$$

It can be noticed that the formula in Equation (2) is similar to the polarization conversion efficiency. The polarization conversion efficiency is defined as the ratio between the output *E_z_* field and the input *E_y_* field, which will show an inverse proportional tendency with the *IL*. Due to the mechanism of the half-wave plate, the electric field can be almost totally converted to the target component except for the energy loss. The output electric fields in the other two components (i.e., *E_x_* and *E_y_*) are confirmed as one order smaller than the target component *E_z_*. Therefore, we discuss *L*_π_ with *IL* in the following *θ* = 45° structures.

Each line in Figure 3b,c represents a different *DC* value, and *DP* is the horizontal axis of this graph. For *θ* = 45°, the smallest value of *L*_π_ (i.e., 2.08 μm) appeared when *DC* = 0.8, *DP* = 0.55, and *DW* = 0.55. The smallest *IL* (i.e., 0.43 dB) appeared when *DC* = 0.4, *DP* = 0.4, and *DW* = 0.46. If considering the applications in quantum communications, such a low *IL* device will be needed. According to the acceptable loss in the system, the relation shown in Figure 3b,c can provide several choices.

Figure 3b reveals that, at different *DC* values, the minimum value of *L*_π_ appeared when 0.5 < *DP* < 0.6, with corresponding *DW* values falling in the range of 0.50–0.65. The trend in Figure 3c reveals that the *IL* increased with the *DP* value. If the *DP* value was between 0.5 and 0.6, the smallest *L*_π_ value was obtained, and the *IL* was not too high. When *DC* increased, *L*_π_ decreased; however, the rate of decrease of *L*_π_ reduced when *DC* > 0.5. As displayed in Figure 3c, an increase in the *DC* value resulted in an increase in the *IL* because an increase in the *DC* value increased the size of the air cubes and caused energy dissipation into air. In summary, the *DC* value must be controlled within a certain range to shorten *L*_π_ without increasing the *IL* substantially.

For *DP* values of 0.5–0.6, the *L*_π_ values of each structure were very close to each other. Therefore, suitable geometry parameters could be selected within an appropriate *L*_π_ range to reduce the *IL*. For example, when *DP* = 0.6 and *DC* > 0.5, the required polarization rotation length (*L*_π_) of each element was approximately 2.19 μm. A structure with *DC* = 0.5 had a low *IL* of 1.6 dB.

**Figure 4:**
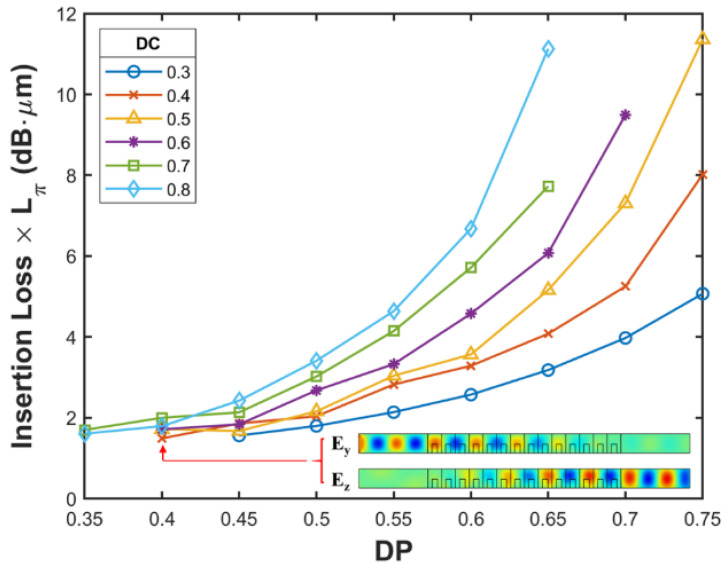
Product of the polarization rotation length and *IL* when *θ* = 45° (the inset depicts the electric field distribution at the minimum value of this product).

Finally, to find the optimal result among many parameter combinations, we plotted the product of the *IL* and *L*_π_ (Figure 4) and determined the combination corresponding to the smallest *IL* and shortest conversion length. Figure 3c and Figure 4 exhibit similar trends, with the overall performance being better when the *DC* and *DP* values were small. However, the minimum value of the aforementioned product (1.49) appeared when *DC* = 0.4, *DP* = 0.4, and *DW* = 0.46 (indicated by the arrow), which indicates that a structure with a smaller *DC* value should be selected to reduce the *IL* to the greatest extent allowed by *L*_π_. Similarly, structures with smaller *DP* and *DW* values exhibit lower *IL*. The inset in Figure 4 illustrates the distribution of the electric field at the point indicated by the arrow, and the inset reveals that the *E_y_* field in this structure (*DC* = 0.4, *DP* = 0.4, and *DW* = 0.46) was completely converted into an *E_z_* field after passing through the photonic crystal wave plate, which confirmed the effectiveness of this structure.

## 4. Conclusions

In this study, we designed a waveguide-type waveplate based on one-dimensional photonic crystals and created a tooth-shaped waveguide on one side of a square waveguide with an air cube array. On the basis of the birefringence of the half-wave-plate, we used the band structure diagram to analyze the polarization rotation length of the PhC-type wave plate structure. We then verified the calculation results through the frequency–domain simulation of the full structure of the wave plate. By using the electric field data for the eigenmode, we calculated the deviation angle of the optical axis of the crystal. We found that when *DP* < 0.4, the *DW* values required to maintain a fixed rotation angle were all very close. This enhances the error tolerance in the fabrication process. By analyzing the effect of variations in the geometric parameters of the air cubes on *θ*, we found that structures with different geometric parameters at *θ* = 45° can have an *L*_π_ value of less than 3.5 μm and an *IL* of less than 5 dB. The product of *L*_π_ and *IL* confirmed that the designed photonic crystal wave plate exhibits superior overall performance when the air cubes have a smaller volume. When designing components, the required parameters can be effectively selected according to the *L*_π_ and *IL* values provided by us. The proposed structure can be fabricated by two steps of E-Beam lithography and inductively coupled plasma dry etching processes. For the first step, the patterns of air squares can be defined by E-Beam lithography and etched by dry etching. For the second step, the waveguides’ pattern can be defined by E-Beam lithography with alignment key and etched by dry etching process. According to the *L*_π_ or *IL* relations as a function of DP, DW, and DC shown above, the geometric parameters of the air cube can provide the fabrication tolerance to a certain extent. Finally, by adjusting the included angle of the optical axis of the wave plate structure, this structure can be used in designing waveguide-type polarization rotation devices.

## Figures and Tables

**Table 1:** Structural parameters when *θ* = 45° and $L_{\pi }<3.5$ μm.

		**DP**	0.35	0.40	0.45	0.50	0.55	0.60	0.65	0.70	0.75
	**DW**										
**DC**											
0.3					0.50	0.55	0.60	0.63	0.67	0.73	0.77
0.4				0.46	0.50	0.55	0.58	0.63	0.67	0.72	0.76
0.5				0.45	0.50	0.54	0.57	0.61	0.66	0.70	0.75
0.6				0.45	0.50	0.53	0.56	0.61	0.65	0.70	
0.7			0.43	0.45	0.49	0.53	0.57	0.61	0.65		
0.8			0.43	0.45	0.48	0.52	0.55	0.60	0.65		

## Data Availability

Not applicable.
